# Causations of phylogeographic barrier of some rocky shore species along the Chinese coastline

**DOI:** 10.1186/s12862-015-0387-0

**Published:** 2015-06-15

**Authors:** Jie Wang, Ling Ming Tsang, Yun-Wei Dong

**Affiliations:** State Key Laboratory of Marine Environmental Science, College of Marine and Earth Sciences, Xiamen University, Xiamen, China; Marine Biodiversity and Global Change Laboratory, Xiamen University, Xiamen, China; Institute of Marine Biology, National Taiwan Ocean University, Keelung, Taiwan

**Keywords:** Freshwater discharge, Rocky shore species, Life history, Ocean current, Population structure, Substrate

## Abstract

**Background:**

Substrate, ocean current and freshwater discharge are recognized as important factors that control the larval dispersal and recruitment of intertidal species. Life history traits of individual species will determine the differential responses to these physical factors, and hence resulting in contrasting phylogeography across the same biogeographic barrier. To determine how these factors affect genetic structure of rocky shore species along the China coast, a comparative phylogeographic study of four intertidal and subtidal species was conducted using mitochondrial and nuclear DNA by combining new sequences from *Siphonaria japonica* with previously published sequences from three species (*Cellana toreuma*, *Sargassum horneri* and *Atrina pectinata*).

**Results:**

Analysis of molecular variance and pairwise Φ_ST_ revealed significant genetic differences between the Yellow Sea (YS) and the other two marginal seas (East China Sea, ECS and South China Sea, SCS) for rocky-shore species (*S. japonica*, *C. toreuma*, *S. horneri*), but not for muddy-shore species *Atrina pectinata*. Demographic history analysis proved that the population size of all these four species were persistent though the Last Glacial Maximum (LGM, ~20 ka BP). Migration analysis revealed that gene flow differentiated northward and southward migration for these four species. However, the inferred direction of gene flow using alternatively mitochondrial or nuclear markers was contradictory in *S. japonica*.

**Conclusions:**

It is concluded that there is a phylogeographical break at the Yangtze River estuary for the rocky shore species and the causation of the barrier is mainly due to the unsuitable substratum and freshwater discharge. All four intertidal and subtidal species appear to have persisted through the LGM in China, indicating the lower impact of LGM on intertidal and subtidal species than generally anticipated. The imbalanced gene flow between YS and ESCS groups for these four species could be explained by historical refugia. The discordance between mitochondrial and nuclear markers in the MIGRATE analysis of *S. japonica* prove the importance of employing multi-locus data in biogeographic study. Climate change, land reclamation and dam construction, which are changing substrate and hydrological conditions around Yangtze River estuary, will consequently affect the biogeographic pattern of intertidal species.

**Electronic supplementary material:**

The online version of this article (doi:10.1186/s12862-015-0387-0) contains supplementary material, which is available to authorized users.

## Background

Phylogeographical patterns of marine faunas are complex and affected by multiple biotic and abiotic factors, and it is in the long-term interest of marine ecologists to understand the roles of these factors in determining the distribution and genetic structuring of species. Glacial-interglacial climate fluctuations during the Pleistocene led to changes of sea level [1, 2] and then caused habitat contractions or expansions [3], which impeded gene flow of marine species and resulted in genetic divergence. Following the glacial retreat, demographic expansion occurred in most marine taxa [4]. Postglacial exchanges of propagules may erase the signals of historic isolation. Accordingly, the biology of the species (e.g. dispersal capacity), availability of suitable habitat and ocean current regimes, which determine the contemporary level of gene flow, would significantly contribute to promote, maintain or homogenize the genetic divergence created by glacial periods. Suitable habitat is assumed to have a significant impact on the phylogeographical distribution of coastal species [5–10] .

The impacts of ocean currents on larval dispersal are variable. Sometimes oceanic currents can promote larval dispersal and enrich population connectivity [11–14]. However, converging ocean currents may also pose a potential barrier to gene flow to some extents [15]. Additionally, a river outflow carrying large amount of sediments and freshwater discharge can influence physical and chemical characteristics (e.g. geomorphology, turbidity, salinity, nutrients and dissolved oxygen etc.) of estuarine and coastal water, which control the biotic dynamics in estuary and coastal ecosystems [16]. This has significant impacts on the dispersal of marine taxa [5, 15, 17–20].

The marginal seas in the northwestern Pacific have changed dramatically in area and configuration, particularly during the Pleistocene glacial-interglacial cycles [21]. Three of the marginal seas, namely the South China Sea (SCS), the Yellow Sea (YS) plus the East China Sea (ECS), and the Sea of Japan (SOJ), served as three independent glacial refugia and resulted in vicariant divergence in marine fauna [22–24]. On the other hand, the lack of genetic structuring in some sympatrically distributed taxa was attributed to their postglacial colonization of these regions [24]. Yet, these hypotheses were not rigorously tested using comprehensive phylogenetic data because most of the studies performed in the region were based on a single marker (in most case the mitochondrial DNA) and largely biased to commercial species, of which the effect of anthropogenic introduction for aquaculture purposes was unknown. Hence, additional investigation using both mitochondrial and nuclear markers was advocated [24].

**Fig. 1:**
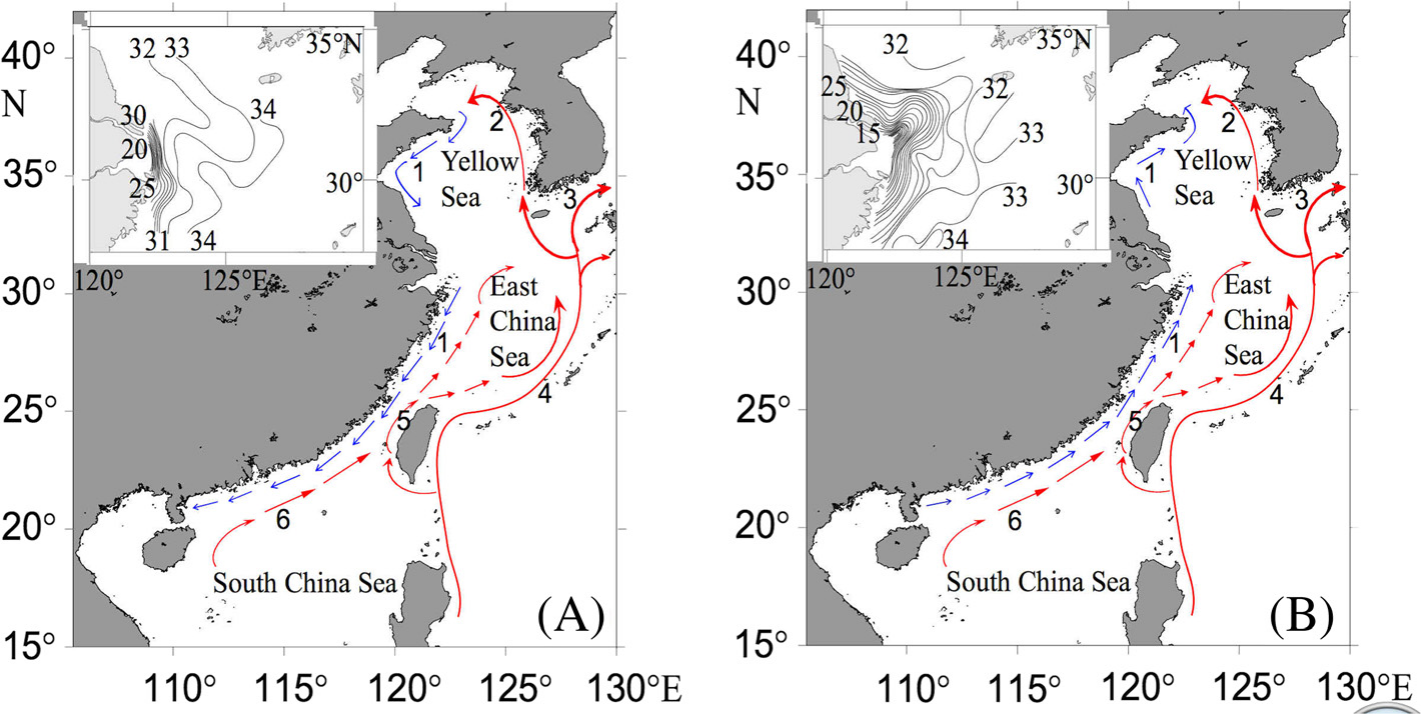
Maps of the coastal currents and sea surface salinity of the Yangtze River estuary (Insert). **a** In winter, the China Coastal Current (CCC) flows from north to south along the China coast. **b** In spring and summer, the (CCC) flows northward into East China Sea (ECS) and turns eastward parallel with the Taiwan Warm Current. 1: China Coastal Current; 2: Yellow Sea Warm Current; 3: Tsushima Warm Current; 4: Kuroshio Current; 5: Taiwan Warm Current; 6: South China Sea Warm Current. Inset: Sea surface salinity of the Yangtze River estuary in winter (**a**) and summer (**b**) [30]

The Yangtze River represents another critical factor in the gene flow of coastal species in China. The Yangtze River, the fifth largest river in the world in terms of volume discharge, brings huge amounts of water (8 ~ 9 × 10^11^ m^3^) into the East China Sea annually [25]. When the freshwater discharge reaches its maximum in spring and summer, Yangtze River plume can extend eastward or northeastward to the Jeju Island (126°08′ ~ 126°58′E, 33°08′ ~ 33°60′N) [26, 27], and it can dramatically change surrounding ocean currents [26, 28] and salinity of the upper layer of the Kuroshio Current [29, 30] (Fig. 1). Moreover, sediment discharges from the Yangtze and other nearby rivers have formed the Yangtze River Delta with an area of more than 3 × 10^4^ km^2^ [31, 32]. The ~600 km long coastline from Lianyungang, Jiangsu Province (34°36′N, 119°13′E) to Shaoxing, Zhejiang Province (30°19′N, 120°46′E) is mainly salt marsh [33]. This together with the Yangtze River discharge is assumed to form a contemporary dispersal barrier for marine species that require a hard substratum (e.g. intertidal rocky shore) or with larvae that cannot tolerant decreased salinity, including the gastropod *Cellana toreuma* [20], the bivalve *Cyclina sinensis* [34] and the macroalga *Sargassum hemiphyllum* [18, 35]. However, studies on other coastal fauna did not detect any genetic split across the Yangtze River [24]. Therefore, more comprehensive study is required to test for the influence of this barrier.

To detect causations (such as substrate, freshwater discharge, coastal current and historical events) of phylogeographic break of intertidal and subtidal rocky shore communities, a multi-species genetic analysis of several intertidal and subtidal species along the Chinese coast was performed. We combined new sequences with previously published sequences from the Chinese coast to construct a database of mitochondrial and nuclear DNA from four species, including three rocky-shore species (limpet *Siphonaria japonica*, limpet *C. toreuma* [20], macroalga *S. horneri* [36]) and one muddy-shore species (bivalve *Atrina pectinata* [37]). These species possess different habitat preferences, reproductive seasons and breeding modes (See Table 1, [37–40]), which provide insights into the responses of intertidal and subtidal species to multi-factors.

**Table 1:** Summary of life history features of four intertidal and subtidal species. The life history features include period of spawning, larval type and larval duration for four intertidal species

Species	Habitat	Spawning period	Larval type	Larval duration	Reference
*Siphonaria japonica*	Intertidal rocky shore	Mar-Jul	Egg ribbons + Pelagic larvae	>7 days	[38]
*Cellana toreuma*	Intertidal rocky shore	Jun-Sep/Oct	Pelagic larvae	4-17 days	[20, 39]
*Sargassum horneri*	Intertidal and subtidal rocky shore	Mar-May	Floating oospores	—	[40]
*Atrina pectinata*	Muddy and sandy shore	May-Oct	Pelagic larvae	17-21 days	[37]

## Results

### Sequence variations

#### Siphonaria japonica

High levels of haplotype diversity (*h*, mean ± S.D.) could be observed in all nine locations of *Siphonaria japonica* for both markers (*COI*, 0.902 ± 0.052 ~ 0.989 ± 0.019; *ITS*, 0.986 ± 0.016 ~ 1.000 ± 0.009; Table 2). The difference in haplotype diversity between four locations from the Yellow Sea (YS) and five locations from the East China Sea (ECS) and the South China Sea (SCS) was statistically significant in *COI* gene (*P* < 0.05), but not in *ITS* sequences.

**Table 2:** Sampling sites and summary of diversity indices of *Siphonaria japonica*. For each locality, individual numbers (n), haplotype diversity (*h*) and nucleotide diversity (*π*) for both mitochondrial and nuclear DNA are listed

Sampling Localities	Abbr.	*COI*			*ITS*		
		n	*h* (Mean ± S.D.)	*π* (Mean ± S.D.)	n	*h* (Mean ± S.D.)	*π* (Mean ± S.D.)
Yellow Sea							
Weihai	WH	25	0.963 ± 0.029	0.0085 ± 0.0047	30	0.986 ± 0.016	0.0067 ± 0.0036
Qingdao	QD	20	0.989 ± 0.019	0.0092 ± 0.0051	20	1.000 ± 0.016	0.0094 ± 0.0050
Rizhao	RZ	20	0.984 ± 0.021	0.0077 ± 0.0044	23	0.996 ± 0.014	0.0088 ± 0.0046
Lianyungang	LYG	25	0.943 ± 0.037	0.0098 ± 0.0054	26	0.988 ± 0.016	0.0075 ± 0.0040
East China Sea							
Ningbo	NB	32	0.944 ± 0.035	0.0079 ± 0.0044	29	1.000 ± 0.009	0.0073 ± 0.0039
Xiamen	XM	28	0.902 ± 0.052	0.0050 ± 0.0030	23	1.000 ± 0.013	0.0095 ± 0.0050
Dongshan	DS	28	0.944 ± 0.037	0.0080 ± 0.0044	27	1.000 ± 0.010	0.0065 ± 0.0035
South China Sea							
Hongkong	HK	37	0.929 ± 0.036	0.0075 ± 0.0042	30	0.998 ± 0.009	0.0072 ± 0.0038
Haikou	HAK	28	0.942 ± 0.037	0.0082 ± 0.0045	25	1.000 ± 0.011	0.0069 ± 0.0037

#### Cellana toreuma, Sargassum horneri and Atrina pectinata

Dong et al. [20] had suggested that the two populations of *C. toreuma* from YS have significant higher haplotype and nucleotide diversity as compared to the populations from ECS and SCS. Re-analysis of published data of *S. horneri* revealed that the difference between populations from YS and populations from ECS/SCS was significant in nucleotide diversity (*P* = 0.034), but not in haplotype diversity (*P* = 0.187). For *A. pectinata*, however, there were no significant difference between populations from YS and populations from ECS in both haplotype diversity and nucleotide diversity.

### Population structure

#### Siphonaria japonica

The global Φ_ST_ analysis of mtDNA and nuclear DNA in *S. japonica* exhibited values of Φ_ST_ significantly different from zero (Additional file 1: Table S1), indicating a significant spatial genetic structure. Pairwise Φ_ST_ analysis of *COI* and *ITS* sequences among locations showed that significant genetic differentiation (*P* < 0.05) between locations from the YS and locations from the ECS and SCS (Table 3). Pairwise Φ_ST_ values were also calculated among different groups. The YS group was significantly different from the ECS group and the SCS group, and the difference between the ECS group and the SCS group was low and non-significant (Table 4), which is similar to the results obtained from *C. toreuma* [20].

**Table 3:** Pairwise genetic distances (Φ_ST_) among locations of *Siphonaria japonica*. Pairwise Φ_ST_ values for nuclear sequence *ITS* and mitochondrial sequence *COI* of *Siphonaria japonica* are given in the upper and lower diagonals, respectively. *P < 0.05; **P < 0.01, ***P < 0.001

	WH	QD	RZ	LYG	ZS	XM	DS	HK	HAK
WH		0.0066	−0.0084	−0.0149	0.0927***	0.0873***	0.0949***	0.1007***	0.1021***
QD	−0.0123		−0.0055	0.0035	0.0339*	0.0273*	0.0336*	0.0285*	0.0290*
RZ	0.0149	−0.0092		−0.0136	0.0629***	0.0452**	0.0598***	0.0599***	0.0663***
LYG	−0.0085	−0.0207	0.0017		0.0914***	0.0699***	0.0817***	0.0905***	0.0946***
ZS	0.2546***	0.2604***	0.3136***	0.2287***		0.0076	0.0002	0.0029	0.0018
XM	0.3846***	0.3989***	0.4623***	0.3585***	0.0084		−0.0028	0.0063	0.0099
DS	0.2577***	0.2709***	0.3191***	0.2370***	−0.0111	0.0091		0.0024	0.0129
HK	0.2758***	0.2858***	0.3383***	0.2541***	−0.0120	−0.0011	−0.0094		−0.0017
HAK	0.2713***	0.2817***	0.3311***	0.2527***	−0.0103	0.0029	−0.0135	−0.0046

**Table 4:** Pairwise genetic distances (Φ_ST_) among groups for each species. The locations of each intertidal and subtidal species were divided into Yellow Sea (YS), East China Sea (ECS) and South China Sea (SCS) along the China Coast. Reference: *Cellana toreuma*, Dong et al. [20]; *Sargassum horneri*, Hu et al. [36]; *Atrina pectinata*, Liu et al. [37].*P < 0.05; ***P < 0.001

Rocky shore species									Muddy shore species	
	*Siphonaria japonica*				*Cellana toreuma*		*Sargassum horneri*		*Atrina pectinata*	
	*COI*		*ITS*		*COI*		*COIII*		*COI*	
	YS	ECS	YS	ECS	YS	ECS	YS	ECS	YS	ECS
ECS	0.3047***		0.0686***		0.1898***		0.0555***		−0.0001	
SCS	0.2830***	−0.0066	0.0761***	0.0051	0.1341***	0.0050	0.0363*	0.0302		

We tested the hypothesis of reduced gene flow between YS and ECS/SCS as observed in pairwise Φ_ST_. Therefore, the locations of the four species were subdivided into two groups (YS group including locations from Yellow Sea and ESCS group containing locations from East and South China Seas) for AMOVA analyses. The hierarchical analysis of AMOVA for *S. japonica* indicated that variation within locations (Φ_ST_) accounted for 70.09 % (*P* < 0.001) and 93.10 % (*P* < 0.001) for *COI* and *ITS* respectively, followed by variation among groups (Φ_CT_) (*CO1*: 30.27 %, *P* = 0.005; *ITS*: 6.87 %, *P* = 0.007) (Table 5). The variations among locations within groups were −0.36 % (*P* = 0.827) for *COI* and 0.03 % (*P* = 0.422) for *ITS* (Table 5).

**Table 5:** Results from analysis of molecular variance (AMOVA) in four intertidal and subtidal species. For each species, locations were divided into two groups (Yellow Sea group and East plus South China Seas group) according to the Yangtze River Estuary. Reference: *Cellana toreuma*, Dong et al. [20]; *Sargassum horneri*, Hu et al. [36]; *Atrina pectinata*, Liu et al. [37]. **P < 0.01, ***P < 0.001

Species	marker	Among groups			Among locations within groups			Within locations		
		d.f.	ΦCT	% Var	d.f.	ΦSC	% Var	d.f.	ΦST	% Var
Rocky shore										
*Siphonaria japonica*										
*COI*		1	0.30265**	30.27	7	−0.00514	−0.36	234	0.29907***	70.09
*ITS*		1	0.06875**	6.87	7	0.00028	0.03	224	0.06901***	93.10
*Cellana toreuma*										
*COI*		1	0.19393**	19.39	13	−0.00259	−0.21	403	0.19185***	80.82
*Sargassum horneri*										
*COIII*		1	0.00367	0.37	5	0.15198***	15.14	100	0.15510***	85.49
Muddy shore										
*Atrina pectinata*										
*COI*		1	0.00391	0.39	6	−0.01297	−1.29	129	−0.00901	100.90

#### *Cellana toreuma*, *Sargassum horneri* and *Atrina pectinata*

One-group AMOVA indicated that the genetic variation among all samples was negative and insignificant in *A. pectinata* (Φ_ST_ = −0.01), but positive and significant in *C. toreuma* and *S. horneri* (Additional file 1: Table S1). At location level, re-analysis of pairwise Φ_ST_ showed that there were no significant differences between all eight locations in *A. pectinata* (Additional file 2: Table S2). However, there existed significant difference between locations from YS and locations from the ECS and SCS (except for FJ location) (Additional file 3: Table S3) in *S. horneri*. At group level, the YS group was significantly different from the ECS group and the SCS group and the difference between the ECS group and the SCS group was low and non-significant in *S. horneri* (Table 4). However, there was no significant difference between the YS group and the ECS group for *A. pectinata* (Φ_ST_ = −0.0001, *P* = 0.436, Table 4).

Under the grouping criteria mentioned above, AMOVA analysis for *COI* of *C. toreuma* showed that there were significant genetic variations among groups (19.39 %, *P* < 0.001) and within locations (80.82 %, *P* < 0.001) (Table 5). AMOVA analysis for *COIII* of *S. horneri* showed significantly high variations among locations within groups (15.14 %, *P* < 0.001) and variation within locations (85.49 %, *P* < 0.001). The variance among groups was relatively low and insignificant (0.37 %, *P* = 0.331; Table 5). For *COI* sequences of *A. pectinata*, variance components at all three levels were non-significant (Table 5).

### Phylogenetic analysis

#### Siphonaria japonica

**Fig. 2:**
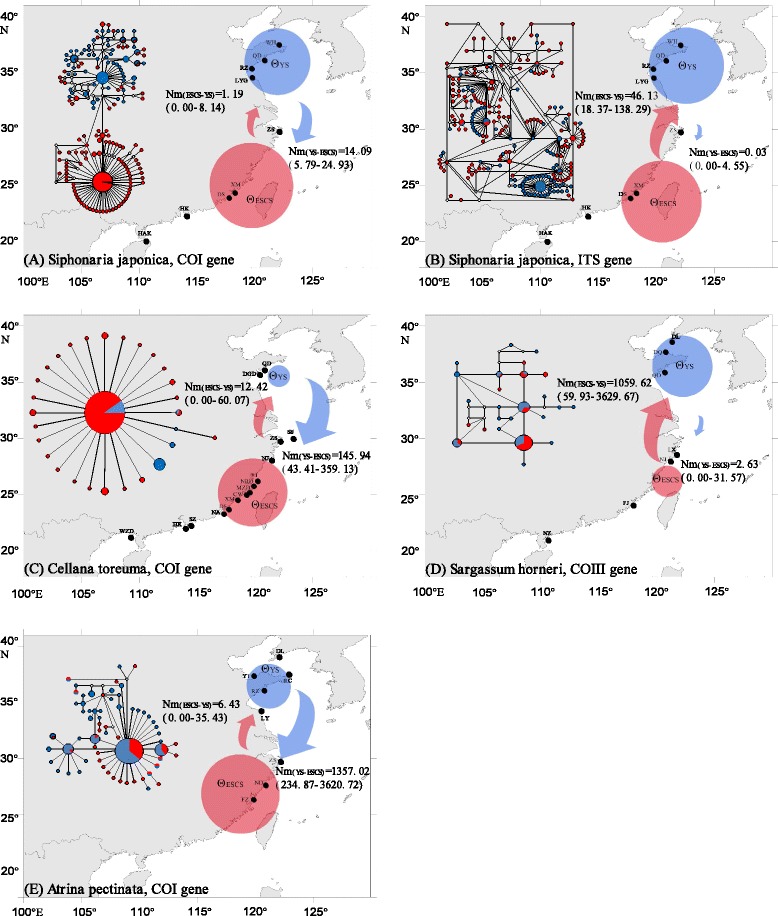
Maps of gene flow and median-joining network (Insert) for each studied species. Map of the China coast showing sampling sites and estimated number of migrants per generation between groups (*Nm*) for each species. All sampling sites of each species are divided into Yellow Sea (YS) group and East plus South China Seas (ESCS) group according to the Yangtze River Estuary. The black dots represent sampling localities for each species. The blue circle and red circle represent the relative magnitude of *Θ*_YS_ (YS group) and *Θ*_ESCS_ (ESCS group) respectively. The blue and red arrows represent the direction and relative magnitude of gene flow estimates between groups. Inset: Haplotype network obtained with mitochondrial DNA for each species and nuclear DNA for *S. japonica*. Each circle represents a single haplotype and sizes are proportional to the number of individuals possessing these haplotypes. Haplotypes of each species are divided into the Yellow Sea (YS) group and the East and South China Seas (ESCS) group separated by the Yangtze River estuary. Colors represent group where haplotypes were detected (blue, YS group; red, ESCS group). The small white circles indicate hypothetical missing haplotypes. Reference: *Cellana toreuma*, Dong et al. [20]; *Sargassum horneri*, Hu et al. [36]; *Atrina pectinata*, Liu et al. [37]

Though branches related to geography were not significantly deep, two putative groups were suggested in the median-joining network and in neighbour-joining tree of mtDNA *COI* of *S. japonica* (Fig. 2a; Additional file 4: Figure S1). One group (northern group) contained all individuals except one (WH22) from the Yellow Sea and 33 individuals from the East and South China Seas. The second group (southern group) included the majority of East and South China Seas individuals plus the single specimen from WH (WH22). Based on network and NJ tree, haplotype relationships of *ITS* sequences revealed no significant branches or clusters corresponding to geography (Fig. 2b; Additional file 5: Figure S2).

#### *Cellana toreuma*, *Sargassum horneri* and *Atrina pectinata*

The haplotype networks of other three species (*C. toreuma*, *S. horneri* and *A. pectinata*) showed a pattern which did not exhibit obvious subdivision according to geographical locations (Fig. 2c, d and e).

### Demographic analysis

#### Siphonaria japonica

**Fig. 3:**
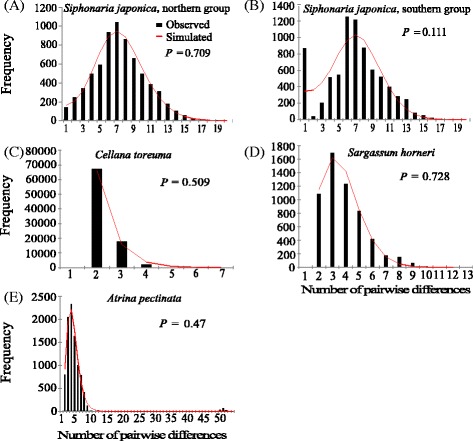
Mismatch distributions of the observed haplotypes for each species. The histograms are the observed frequencies of pairwise divergences and the line refers to the expectation under the sudden population expansion model. All *COI* sequences of *S. japonica* were divided into two putative groups: northern group and southern group, according to the phylogenetic analyses (See the results in the phylogenetic analysis). Reference: *Cellana toreuma*, Dong et al. [20]; *Sargassum horneri*, Hu et al. [36]; *Atrina pectinata*, Liu et al. [37]

**Fig. 4:**
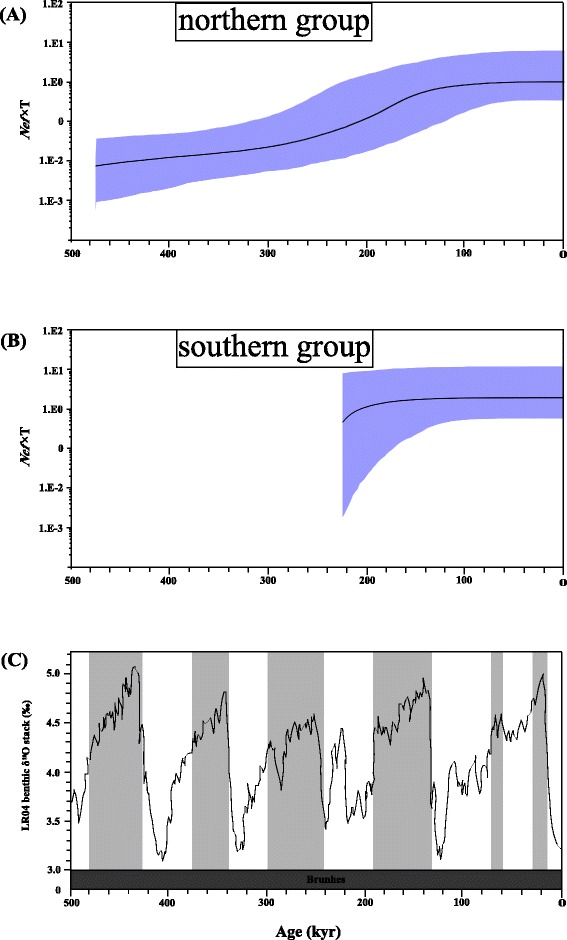
Bayesian skyline plots (BSP) for *Siphonaria japonica*. All *COI* sequences of *S. japonica* were divided into two groups: northern group (**a**) and southern group (**b**), according to the phylogenetic analyses (See the results in the phylogenetic analysis). The black line represents median population estimates; the upper and lower limits of light blue shading represent the 95 % confidence intervals. The gray rectangles represent the glacial stages of various oxygen isotope stages [42, 43] in (**c**)

Mismatch distribution analysis showed a unimodal distribution for both northern group and southern group of *COI* in *S. japonica*, suggesting rapid demographic expansion (Fig. 3a and b). The tau value (*τ*) can provide a rough estimation of the time when rapid population expansion began. Using a substitution rate of 1 % per million years [9, 41], the time of demographic expansion for northern group and southern group was estimated as ~440 ka (90 % CI: 313.9-682.4 ka) BP and ~190 ka (90 % CI: 104.4-259.3 ka) BP, respectively (Table 6). The BSPs indicated that the northern group had a slightly increase of population size since 475 ka BP, but the most distinct demographic expansion happened around 200 ka BP (Fig. 4a). The southern group exhibited a gentle growth in population size from ~225 ka BP (Fig. 4b), when the earth was at an interglacial stage (Fig. 4c, [42, 43]). The inconsistent estimates of demographic expansion could be the difference between modeling an expansion with pairwise differences in the mismatch distribution analysis versus the coalescent theory in the BSP analysis.

**Table 6:** Results of population expansion tests on the four species. Fu’s *F*_*S*_, Tajima’s *D* and mismatch distribution estimate (*τ*) and the real expansion time (*t*) with 90 % credibility intervals in parentheses for each species are included. All *COI* sequences of *S. japonica* were divided into two groups: northern group and southern group, according to the phylogenetic analyses (See the results in the phylogenetic analysis). Reference: *Cellana toreuma*, Dong et al. [20]; *Sargassum horneri*, Hu et al. [36]; *Atrina pectinata*, Liu et al. [37]. *P < 0.05; **P < 0.01, ***P < 0.001

Gene		Neutrality test		Mismatch distribution	
		Fu’s *F*_*S*_	Tajima’s *D*	*τ*	*t* (ka)
*Siphonaria japonica*					
*COI*	northern group	−25.029***	−2.199*	5.750 (4.094-8.898)	440.9 (313.9-682.4)
	southern group	−26.964***	−2.781***	2.486 (1.361-3.381)	190.6 (104.4-259.3)
*Cellana toreuma*					
*COI*		−30.175***	−2.497***	3.000 (0.422-3.213)	502.5 (70.7-538.2.)
*Sargassum horneri*					
*COIII*		−18.353***	−1.531*	1.875 (0.75-3.072)	119.3 (47.7-195.5)
*Atrina pectinata*					
*COI*		−26.277***	−2.527***	1.648 (1.295-4.086)	170.1 (133.7-421.7)

A posterior simulation-based analogue of Akaike’s information reiteration through MCMC (AICM) test for northern group revealed that the expansion model favored over the other two models (Additional file 6: Table S4), and the BSP showed a demographic expansion. The constant size model was the best-fitting demographic model for southern group (Additional file 6: Table S4) which exhibited a similar pattern to the BSP.

#### *Cellana toreuma*, *Sargassum horneri* and *Atrina pectinata*

Although mitochondrial sequences in both *C. toreuma* and *S. horneri* showed a significant value of Φ_ST_, we reconstructed their demographic history with mismatch distribution and BSP because, no spatial subdivision of haplotypes of mitochondrial sequence existed in the network (Fig. 2c, d). The BSP analysis showed that the population size of *C. toreuma* had an extremely gentle increase since 260 ka BP (Additional file 7: Figure S3A), and the population size of *S. horneri* experienced a very slow increase since 194 ka BP with the mutation rate of 1.675 % MY^−1^ (Additional file 7: Figure S3B). The population size of *A. pectinata* increased significantly since 232 ka BP with the mutation rate of 0.775 % MY^−1^ (Additional file 7: Figure S3C). Mismatch distribution analysis showed that the shapes of mismatch distribution were unimodal (Fig. 3c, d, e) and the time of demographic expansion for *C. toreuma*, *S. horneri* and *A. pectinata* were 502.5 ka (90 % CI: 70.7-538.2 ka) BP, 119.3 ka (90 % CI: 47.7-195.5 ka) BP and 170.1 ka (90 % CI: 133.7-421.7 ka) BP respectively (Table 6). Therefore, the demographic expansion of the two groups of *S. japonica* and other three species significantly predated the Last Glacial Maxima (~20 ka BP).

AICM tests for *C. toreuma* and *S. horneri* showed that the constant size model was a better fit to the data than the BSP and expansion models (Additional file 6: Table S4). It was not surprising for these two rocky-shore specie as they both showed relatively flat BSPs. The expansion model was strongly favored for *A. pectinata* that showed a significant demographic expansion in BSP.

### Gene flow

#### Siphonaria japonica

Because the present study mainly focused on the gene flow across the Yangtze River Estuary, locations of each species were divided into YS group and ESCS group to detect the possible phylogeographical break. In the present studies of *S. japonica*, contrasting patters of gene flow between mitochondrial and nuclear markers were suggested (Fig. 2a and b, Additional file 8: Table S5). For the mitochondrial sequences, there is southward gene flow from YS to ESCS (*Nm*_(YS-ESCS)_ = 5.79-24.93), but little gene flow northwards from ESCS to YS (*Nm*_(ESCS-YS)_ = 0.00-8.14). The *ITS* analyses, however, indicated there was almost no gene flow in a southward direction from YS to ESCS (*Nm*_(YS-ESCS)_ = 0.00-4.55), compared with a high gene flow from ESCS to YS (*Nm*_(ESCS-YS)_ = 18.37-138.29).

#### *Cellana toreuma*, *Sargassum horneri* and *Atrina pectinata*

Re-analysis of published data showed that significant higher southward gene flow could be observed in *C. toreuma* (*Nm*_(YS-ESCS)_ = 43.41-359.13, Fig. 2c) and *A. pectinata* (*Nm*_(YS-ESCS)_ = 34.87-3620.72, Fig. 2e). On the contrary, there is almost no gene flow in a southward direction (*Nm*_(YS-ESCS)_ = 0.00-31.57, Fig. 2d) in *S. horneri*, while large gene flow can be observed from ESCS to YS (*Nm*_(ESCS-YS)_ = 59.93-3629.67).

## Discussion

### Phylogeography in intertidal and subtidal species along the China coast: northern and southern differentiation

Significant population differentiation existed among northern (Yellow Sea) and southern groups (East China Sea and South China Sea) of *Siphonaria japonica, Cellana toreuma* and *Sargassum horneri* respectively, indicating that there is a genetic break between northern and southern populations of rocky-shore species. For mitochondrial and nuclear sequences of *S. japonica*, pairwise Φ_ST_ analyses among different locations indicated that Yellow Sea locations were significantly different from locations from the East China Sea and the South China Sea (Table 3). When these locations were divided into three groups (YS, ECS and SCS) based on their geographic locations, the Φ_ST_ values between the YS group and the other two groups (ECS and SCS) were significant for limpet *S. japonica* and macroalga *S. horneri*. This result is similar to results for the limpet *C. toreuma*, and suggests that the Yellow Sea group is relatively isolated from the other two groups. AMOVA analyses for *COI* and *ITS* of *S. japonica* and for *COI* of *C. toreuma* showed that genetic differentiation among YS and ESCS groups accounted for a high proportion of the molecular variance among groups (Table 5), which suggested that populations were mainly grouped according to the Yangtze River Estuary. Phylogenetic analysis showed that two putative groups existed in *COI* of *S. japonica*, which also demonstrated a genetic break around the Yangtze River Estuary. A geographic genetic break can also be detected by changes in gene diversity between populations. Significant difference in genetic diversity between locations from YS and locations from ECS/SCS for these three rocky-shore species supported the suggestion that a genetic break existed between YS and ECS/SCS groups.

In contrast to the rocky-shore species, no obvious population structure for the muddy-shore species (*Atrina pectinata*) was found based on the results from AMOVA analysis and pairwise Φ_ST_ values. The absence of genetic difference between YS and ECS groups as detected for the muddy-shore species was similar to previous studies of *Rapana venosa* [44], *Tegillarca granosa* [45] and *Cyclina sinensis* [46].

During the Pleistocene glacial-interglacial cycles, areas and configurations of the marginal seas have changed dramatically in the West Pacific [21, 47]. When the sea level fell about 120-140 m over the past ~ 800 kyr [48], the Yellow Sea (YS) and the East China Sea (ECS) were reduced to an elongated trough, the Okinawa Trough, and the South China Sea (SCS) became a semi-enclosed gulf [49]. ECS and SCS were separated by a land bridge which connected Taiwan and the continent [50]. Previous comparative phylogeographical studies in marginal seas of the northwestern Pacific have suggested that historical isolation between SCS and ECS plays important roles for the present-day distribution of genetic variation of coastal species such as some fishes, crustaceans and muddy/sandy shore mollusks [24].

### Multi-factors controlling the population genetic differentiation of rocky-shore species

The contrasting phylogeographical patterns between rocky and muddy intertidal species indicates that substrate plays important roles in the phylogeographical patterns of intertidal species. From Lianyungang, Jiangsu Province to Shaoxing, Zhejiang Province, the absence of appropriate habitats (~600 km salt marsh shore) could hamper the settlement of rocky intertidal species and consequently genetic exchange between Yellow Sea and East China Sea populations. The negative impacts of a lack of appropriate substrate on genetic connectivity have been reported in other intertidal fauna. For instance, the existence of long stretches of sandy beach serves as a barrier to dispersal between Cape St Lucia and Zinkwazi Beach in the limpet *S. nigerrima* in southeast Africa [9]. Recently, a genetic analysis of *S. japonica* specimens collected from Yangguang Island (32°36′N, 121°08′E), an artificial island between Zhoushan (30°01′N, 122°06′E) and Lianyungang (34°36′N, 119°13′E), was carried out and revealed that this location is genetically similar to the location in Zhoushan. These results indicate that colonization of some rocky intertidal species can happen across the barrier if suitable habitat is provided (Huang XW, Wang W and Dong YW, unpublished observations). In contrast, as seen above, the species living in muddy substrate appear unaffected by the salt marsh around at the Yangtze River estuary.

Freshwater discharge influences hydrological condition nearby estuary and may have some impacts on gene flow between the Yellow Sea and East China Sea. During spring and early summer, the spawning season of *S. japonica*, the Taiwan Warm Current (TWC) and the China Coast Current (CCC) in ECS and SCS flowing northward [51] transport pelagic larvae from SCS to ECS. However, the freshwater discharge from the Yangtze River will cause deflection of the East China Sea coastal current. The size and distance of deflection is more prominent in spring and summer due to the increased amount of surface runoff in the rainy season [52]. In spring and summer, the plume of water from the Yangtze River discharge shifts in a northerly direction in parallel with the TWC with a clockwise deflection (Fig. 1b). When it reaches its maximum flow, the Yangtze River discharge affects surrounding hydrological conditions [26, 28] and causes a decline in the salinity of the upper layer of the Kuroshio Current [29], which could influence the northward transport of larvae into the Yellow Sea. Dong et al. [20] also suggested that unique haplotype and higher genetic diversity in YS group were mainly contributed to the ocean current and freshwater discharge during the spawning season of *C. toreuma*. The impact of freshwater discharge on the phylogenetic distribution of marine species has been widely observed in previous studies. For example, the outflow from the Amazon River has been invoked as a major factor for the biogeographic break between Brazilian and Caribbean faunas (e.g. barnacle *Chthamalus proteus* [53] and surgeonfishes *Acanthuridae sp* [5]).

Although the same phylogeographic break around the Yangtze River estuary was observed for two rocky intertidal limpets (*C. toreuma* [20]; *S. japonica*, the present study), haplotype networks of these two limpets were different. The haplotype network of *C. toreuma* is a single star-like network [20] and *S. japonica* presents a relatively complex network with two putative groups (Fig. 2). *C. toreuma* lays its eggs into sea water directly and the length of the pelagic larval stage varies between 4–18 days in congenerics [54]. *S. japonica* deposits gelatinous egg ribbons containing numerous small eggs on the rocky shore, and then the eggs develop into planktotrophic veliger larvae within ~15 days. The veliger larvae of *S. japonica* could at least maintain in the plankton about seven days (Wang W and Dong YW, unpublished observations). Therefore, the discrepancy of phylogeographic patterns between *S. japonica* and *C. toreuma* could be associated with their different reproductive modes and larval dispersal capabilities. The high dispersal capability of the brown macroalga *S. horneri* could be partly attributed to low variance among groups based on the AMOVA analysis. *S. horneri* can breed by both sexual reproduction and asexual reproduction, and can form floating mats which can drift about 1–5 months after being detached from the substratum [55–57].

### Historical demography of intertidal and subtidal species along the China coast

Even though the expansion model and the constant size model received strong support over the BSP for the putative northern and southern groups in *S. japonica*, respectively, evidences from the mismatch distribution analysis and the BSP suggested northern group and southern group had different timings of population expansion events. The expansion time of northern group (313.9 ~ 682.4 ka BP) was earlier than that of southern group (104.4 ~ 259.3 ka BP). This corroborates recent evidence of the existence of a northern refugium in the Northwestern Pacific observed in other marine organisms (e.g. seaweed *Ishige okamurae* [58], limpet *C. toreuma* [20] and barnacle *Chthamalus challengeri* [59]). In northwestern Pacific, South China Sea (SCS) and East China Sea (ECS) are widely accepted as southern glacial refugia [22–24]. Thus, new evidences of the existence of a northern refugium in this area may be helpful to illustrate the influence both of past populations in glacial refugia and of contemporary gene flow in shaping current phylogeographical patterns in the future studies. Furthermore, the population sizes of these four species were persistent through the Last Glacial Maximum (LGM; ~20 ka BP). Recent meta-analyses of demographic history of intertidal rocky organisms in the northeastern Pacific [3] and northwestern Pacific [24] also converged on similar finding that majority of the species were not extirpated entirely by the LGM and regional persistence maybe more prevalent.

At the contemporary level, MIGRATE analyses revealed that migration rates were unbalanced between ESCS and YS groups of *S. japonica*. However, the inferred direction of gene flow using alternatively mitochondrial or nuclear markers was contradictory. *COI* analyses suggested that southward migration rate was almost ten times the northward migration rate. *ITS* sequences suggested the northward migration rate was significantly higher (Fig. 2b). Discordances between nuclear and mitochondrial data in animal biogeographic studies are not uncommonly reported [60]. Adaptive introgression, demographic disparity and sex biased migration are commonly invoked as potential explanation of discordance observed [60]. Sex biased gene flow appears unlikely for this hermaphroditic limpet with planktonic larvae transported by ocean current while adaptive introgression is difficult to test based on the present data. The mitochondrial genes have a higher mutation rates and smaller effective population size than the nuclear genes, and hence considered to be more informative in shallow relationships. Besides, the high imbalance between immigration and emigration rates in other three species (Fig. 2c, d and e) could be associated with historical refugia and suggested the likely existence of sources and sinks at metapopulation level. Recent studies emphasize the need of multi-locus data for accurate estimation of various population parameters [3, 61]. As a result, more data are required to determine the underlying mechanism leading to the discordance, and our results emphasize the importance of employing multi-locus data in biogeographic study.

### Biogeography of intertidal species in China

The phylogeographic barrier of rocky intertidal species around Yangtze River estuary will possibly disappear under the coupled impacts of climate change and human activities. Firstly, northward shift of rocky intertidal species is ubiquitous in the scenario of climate change [62–65]. The coastal sea surface temperature (SST) and extreme hot days have continued to increase from 1982 to 2010 [66, 67]. The increasing temperature will potentially force the northward shift of intertidal species along China coast; Secondly, land reclamation has resulted in about 55 % loss of coastal wetland in China from 1949 to 2002 [68], and large numbers of artificial structures provide hard substrates in areas where these are generally absent and act as stepping stones to connect the Yellow Sea populations and East China Sea/South China Sea populations; Finally, about 50,000 dams constructed in the Yangtze River catchment from the late 1950 to 2003 have a storage capacity of 22 % of the annual water discharge (200 × 10^9^ m^3^ in 2003) and result in a strong decrease of sediment and freshwater discharge in spring and summer [69]. The changing hydrological condition around the Yangtze River estuary could enhance the possibility of larval dispersal across it. Overall, the potential northward shift of rocky intertidal species will change the biogeographic pattern along the Chinese coast and adaptive management should be considered for future management of rocky intertidal ecosystem in China.

## Conclusions

A significant phylogeographic break, occurring around the Yangtze River Estuary, was observed for populations of rocky-shore species along the China coast. Substrate, ocean current and freshwater discharge are suggested as major factors determining the contemporary structure of rocky-shore species by limiting north–south dispersal of planktonic larvae. In addition, historical events and life history characteristics can also influence the contemporary phylogeographic distribution of intertidal and subtidal species. However, human activities are changing the habitat of intertidal species. Large-scale land reclamation activities can change the sedimentary substrate, and so provide suitable hard substrates for colonization by rocky-shore species. On the other hand, numerous dams constructed in the Yangtze River catchment decrease the riverine sediment supply to the sea and will impact on the environment of the Yangtze Delta and the nearby coastal ocean.

## Methods

### Sampling and sequencing

A DNA sequence database consisted of new sequences (cytochrome *c* oxidase subunit I gene, *COI*; internal transcribed spacer, *ITS*) from *Siphonaria japonica* (specimens were collected from nine rocky shore localities along the Chinese coastline between May 2012 and January 2013) and previously published data (mitochondrial gene) from three other intertidal and subtidal species in the Pacific Northwest (Fig. 2).

Partial sequences of *COI* and *ITS* in *S. japonica* were amplified by polymerase chain reaction (PCR). *COI* sequences were amplified with universal primers LCO1490 and HCO2198 [70] and *ITS* sequences were amplified using primers *its*-1d and *its*-4r [71]. PCRs were conducted in a 25-μL reaction volume containing 2.5 μL of 10 × buffer (Mg^2+^ Plus), 2 μL of 2.5 mM dNTPs, 1 μL of each 10 mM primers, 0.25 μL (1.25 U) of *Taq* DNA polymerase and 200 ng DNA template. Amplification was initiated with denaturing at 95 °C for 3 min, followed by 35 cycles of 95 °C for 1 min, annealing at 40 °C for *COI* and 54 °C for *ITS* for 1 min and 72 °C for 1 min and then a final extension at 72 °C for 10 min. After visualizing the target amplicon in 1.5 % agarose gels, PCR products of *COI* gene were sent to a commercial company for sequencing (Invitrogen Biotechnology Co., Ltd., Shanghai, China). The *ITS* products were purified using the PCR Purification Kit (Aidlab Biotechnologies Co., Ltd., Beijing, China), ligated to pMD19-T Vector (TaKaRa Biotechnology, Dalian, China) and then transformed into competent cell of *Escherichia coli* DH5α (TaKaRa Biotechnology, Dalian, China). Finally, a positive clone per individual was sequenced in both directions using M13 and M17 primers by Invitrogen Biotechnology Co., Ltd. (Shanghai China).

### Sequence variation and population genetic analysis

All sequences in *Siphonaria japonica* were edited by comparing both strands using DNAMAN 7 software (LynnonBioSoft, Quebec, Canada), and then aligned with MUSCLE [72] using MEGA 5 [73] with default settings. Standard molecular diversity indices including haplotype diversity (*h*) and nucleotide diversity (*π*), and neutrality test including Fu’s *F*_s_ [74] and Tajima’s *D* [75] were calculated using ARLEQUIN 3.5 [76].

jModelTest 2.1.1 [77] was utilized to estimate the best-fitting substitution model and substitution parameters with the Bayesian information criterion (BIC) for each species (Table 3). For sequences from *S. japonica*, phylogenetic analyses were performed based on the neighbour-joining (NJ) approach using MEGA incorporating the Tamura-Nei model (TrN) [78] with corresponding gamma correction for *COI* and *ITS* (the closest model in MEGA to TVM + G is the TrN + G model for NJ tree building). 1 000 bootstrap replicates were carried out to assess the clade credibility of the NJ phylogram. A haplotype network illustrating genealogical relationships between haplotypes was constructed for each species using the median-joining (MJ) method with Network 4.6 [79].

Pairwise Φ_ST_ measures were calculated to evaluate the levels of genetic differentiation and significance was estimated with 10 000 permutations using ARLEQUIN. Furthermore, hierarchical analysis of molecular variance (AMOVA) [80] was performed to test for possible phylogeographic separation. In the present study, genetic differentiation among locations was only analyzed in *S. japonica*, because the published data from other three species have been analyzed. Because this study focused on the gene flow and phylogeographic break, the locations of each species were divided into three groups: the Yellow Sea (YS) group, the East China Sea (ECS) group and the South China Sea (SCS) group based on the geographical locations (refer to Table 4 for the allocation of sites). Because no significantly genetic differentiation was observed between ECS and SCS (see the Results Table 3), the ECS and SCS were combined as the ESCS group to compare with the YS group to test for the hypothesis of reduced gene flow across the Yangtze River outflow. On the other hand, samples from all locations for each species were considered as a single group to verify the significance of partitioning of genetic variance among all samples.

### Historical demography

The coalescent-based approach is widely used to reconstruct the demographic history. In the present study, the Bayesian skyline plot (BSP) was generated in BEAST v1.7.4 [81] to estimate the change in effective population size over time. The analyses were only performed with the mitochondrial dataset because no distinct clade was revealed in *ITS* and the mutation rate is unknown for the marker, hampering the estimate of temporal scale. Analyses were conducted under corresponding model suggested by jModelTest for each species with constant Bayesian skyline tree priors with 10 groups under a strict clock model. Default priors were used for parameter settings. Three independent MCMCMC searches were run for 100 million generations and parameters were recorded every 10 000 generations with the first 10 million generations discarded as burn-in. For each run, the effective sample sizes (ESS) of important parameters sampled from the MCMCMC were >1000 in all three replicate runs calculated by TRACER 1.4 [81]. Results from three runs were then pooled with LOGCOMBINER version 1.7 [81] for final reconstruction of the BSP. Pairwise mismatch distribution analysis was also performed using the mitochondrial dataset of each species with ARLEQUIN 3.5 [76] to validate the result from BSP. Population expansion parameters (*τ*) were transformed to estimates of real-time since expansion (*t*) with the formula *τ* = 2μ*kt*, where μ is the mutation rate and *k* is the sequence length. As two geographically-partitioned groups were revealed in phylogenetic analysis of *COI* in *S. japonica* (Fig. 2a), the demographic history of each putative group was reconstructed using both methods above.

Because of the absence of clear fossil or geological record, Colgan & Costa [41] and Teske et al. [9] had used a mutation rate of 1 % per million years to estimate divergence times in *Siphonaria* genus, when referring to available calibrated fossil data for marine gastropod. Thus, in this study, such a mutation rate (1 % Myr^−1^) with a generation of 1 year was applied for *COI* of *S. japonica*. A divergence rate of 0.85-1.15 % Myr^−1^ for *COI* was set in *C. toreuma*, as used for estimation times of the *C. nigrolineata* [8]. The divergence rate was set at 2.6-4.1 % Myr^−1^ for *COIII* of *S. horneri* [36] and 0.7-2.4 % Myr^−1^ for *COI* of *A. pectinata* [37], respectively, which were proposed in the original papers. In addition a generation of 1 year for *C. toreuma* [39] and *S. horneri* [82] and a generation of 2 years for *A. pectinata* [83] were assumed. In the present study, the mutation rate for these three species (*C. toreuma*, 0.5 % Myr^−1^; *S. horneri*, 1.675 % Myr^−1^; *A. pectinata*, 0.775 % Myr^−1^) was obtained by averaging the divergence rates used above and then dividing by two.

To evaluate whether the BSP was the best model in reconstructing demographic histories, BSP model was compared with two simple moles in Tracer V 1.6: constant population size and expansion growth. The two alternative models were run in BEAST using the same way as mentioned above for BSP. A posterior simulation-based analogue of Akaike’s information criterion through MCMC (AICM) [84] was used to compare all three models, which measured AIC from the posterior of each model, with score > 10 as strong evidence in favor of one model over the others [85].

### Gene flow analysis

To examine the level and direction of contemporary gene flow across the phylogeographic break, we adopted the coalescent-based approach implemented in MIGRATE-N 3.5.1 [61] for estimating the migration rates between groups within the four studied species. Locations were grouped into a “YS” group and an “ESCS” group according to their geographical distribution. Random sub-samples were performed to allow the two groups to contain comparable number of individuals. The Bayesian approach was utilized to infer the mutation-scaled effective population size (*Θ = 2Nμ*, with *N* = effective population size and *μ* = mutation rate) and the mutation-scaled effective immigration rate (*Μ = m/μ*, with *m* = immigration rate) [61]. Analyses were conducted with a full migration matrix model (*Θ* and *Μ* were estimated jointly from the data). The effect number of migrates per generation (*Nm*) among groups can be calculated by multiplying *Θ* and *Μ* together. For the Bayesian approach, a single long chain with slice sampling for the proposal distribution was used according to the recommendation of the author [61]. We performed Migrate with the DNA sequence model. Initially, short runs were performed to estimate *Θ* and *Μ* with F_*ST*_ with a uniform prior for parameters, followed by subsequent runs set using parameters estimated in the short runs. Five independent sets of runs were conducted, each containing one long chain of 5 000 000 steps with a burn-in time of 500 000, a sampling increment of 1000, and an adaptive heating scheme with four chains and temperatures of 1.0, 1.5, 3.0, and 10 000. The results of the independent runs were congruent and the last run was chosen for interpretations.

## Availability of supporting data

The newly obtained DNA sequences: GenBank accessions: KF716505-KF716747 mtDNA; KF716748-KF716980 nuclear gene for *Siphonaria japonica*. The reanalyzed data: *Cellana toreuma* (*COI*, GenBank accession number, JQ313140-JQ313557; [20]), *Sargassum horneri* (*COIII*, GenBank accession number, JF461002-JF461052; [36]), *Atrina pectinata* (*COI*, GenBank accession number, HQ449254-HQ449388; [37]). All the alignments data supporting the results of this article are available in the Dyad data repository under doi: 10.5061/dryad.2mv73 [86].

## Additional files

Additional file 1: Table S1. Estimates of genetic structure among all samples for each species. Reference: *Cellana toreuma*, Dong et al. [20]; *Sargassum horneri*, Hu et al. [36]; *Atrina pectinata*, Liu et al. [37]. Additional file 2: Table S2. Pairwise genetic distance (ΦST) among locations and *P*-values of mitochondrial sequence *COI* of *Atrina pectinata* are given in the lower and upper diagonals, respectively. Reference: *Atrina pectinata*, Liu et al. [37]. Additional file 3: Table S3. Pairwise genetic distance (ΦST) among locations and *P*-values of mitochondrial sequence *COIII* of *Sargassum horneri* are given in the lower and upper diagonals, respectively. Reference: *Sargassum horneri*, Hu et al. [36]. Additional file 4: Figure S1. Neighbour-joining tree of *COI* from *Siphonaria japonica*. Neighbour-joining tree constructed with mitochondrial DNA haplotype data from *Siphonaria japonica*. The classification of the two groups is given on the right. Additional file 5: Figure S2. Neighbour-joining tree of *ITS* from *Siphonaria japonica*. Neighbour-joining tree constructed with nuclear DNA haplotype data from *Siphonaria japonica*. Additional file 6: Table S4. A posterior simulation-based analogue of Akaike’s information reiteration through MCMC (AICM) test was used to compare demographic models for all four species. The estimated AICM scores of the posterior are listed in the third column for each species, and lower value indicates a better fit to the data. Boldface corresponds to the best fitting demographic model. The AICM comparisons were presented in the matrix of columns 5 to 7. The positive value represents the support for the one model over another. Reference: *Cellana toreuma*, Dong et al. [20]; *Sargassum horneri*, Hu et al. [36]; *Atrina pectinata*, Liu et al. [37]. Additional file 7: Figure S3. Bayesian skyline plots (BSP) for *Cellana toreuma* (A), *Sargassum horneri* (B) and *Atrina pectinata* (C). The black line represents median population estimates; the upper and lower limits of light blue shading represent the 95 % confidence intervals. Reference: *Cellana toreuma*, Dong et al. [20]; *Sargassum horneri*, Hu et al. [36]; *Atrina pectinata*, Liu et al. [37]. Additional file 8: Table S5. The effective population size (*Θ*) and the effective immigration rates (*M*) with 97.5 % credibility intervals in parentheses were estimated using the program MIGRATE for each species. All sampling sites of each species are divided into Yellow Sea (YS) group and East plus South China Seas (ESCS) group according to the Yangtze River Estuary. Reference: *Cellana toreuma*, Dong et al. [20]; *Sargassum horneri*, Hu et al. [36]; *Atrina pectinata*, Liu et al. [37].
