# Supplementary material for: Causations of phylogeographic barrier of some rocky shore species along the Chinese coastline
Source: BMC Evol Biol. 2015 Jun 15;15:114. doi: 10.1186/s12862-015-0387-0 (PMC4465721; doi:10.1186/s12862-015-0387-0)
Supplement: Additional file 1: Table S1. — Estimates of genetic structure among all samples for each species. Reference: Cellana toreuma, Dong et al. [20]; Sargassum horneri, Hu et al. [36]; Atrina pectinata, Liu et al. [37]. [file 12862_2015_387_MOESM1_ESM.docx]

**Additional file 1: Table S1.** Estimates of genetic structure among all samples for each species. Reference: *Cellana toreuma*, Dong *et al*. [20]; *Sargassum horneri*, Hu *et al*. [36]; *Atrina pectinata*, Liu *et al.* [37].

| **Species (gene)** | **Φ_ST_** | ***P*** |
| --- | --- | --- |
| *Siphonaria japonica* (COI) | 0.18 | <0.001 |
| *S. japonica* (ITS) | 0.06 | <0.001 |
| *Cellana toreuma* (COI) | 0.05 | <0.001 |
| *Sargassum horneri* (COIII) | 0.15 | <0.001 |
| *Atrina pectinata* (COI) | -0.01 | >0.05 |
