# Supplementary material for: Causations of phylogeographic barrier of some rocky shore species along the Chinese coastline
Source: BMC Evol Biol. 2015 Jun 15;15:114. doi: 10.1186/s12862-015-0387-0 (PMC4465721; doi:10.1186/s12862-015-0387-0)
Supplement: Additional file 2: Table S2. — Pairwise genetic distance (ΦST) among locations and P-values of mitochondrial sequence COI of Atrina pectinata are given in the lower and upper diagonals, respectively. Reference: Atrina pectinata, Liu et al. [37]. [file 12862_2015_387_MOESM2_ESM.docx]

**Additional file 2: Table S2.** Pairwise genetic distance (ΦST) among locations and *P*-values of mitochondrial sequence COI of *Atrina pectinata* are given in the lower and upper diagonals, respectively. Reference: *Atrina pectinata*, Liu *et al*. [37].

|  | **DL** | **YT** | **RC** | **RZ** | **LY** | **ZS** | **ND** | **FZ** |
| --- | --- | --- | --- | --- | --- | --- | --- | --- |
| DL |  | 0.7025 | 0.2047 | 0.6942 | 0.3801 | 0.9692 | 0.2538 | 0.8206 |
| YT | -0.0135 |  | 0.3223 | 0.7734 | 0.8542 | 0.6849 | 0.2027 | 0.8262 |
| RC | 0.0251 | 0.0045 |  | 0.2599 | 0.3596 | 0.2959 | 0.1989 | 0.8849 |
| RZ | -0.0150 | -0.0150 | 0.0158 |  | 0.7745 | 0.9619 | 0.1714 | 0.7143 |
| LY | 0.0029 | -0.0161 | 0.0041 | -0.0159 |  | 0.7977 | 0.1557 | 0.8378 |
| ZS | -0.0257 | -0.0068 | 0.0072 | -0.0272 | -0.0139 |  | 0.4377 | 0.9180 |
| ND | 0.0245 | 0.0454 | 0.0570 | 0.0431 | 0.0519 | 0.0006 |  | 0.3153 |
| FZ | -0.0151 | -0.0088 | -0.0303 | -0.0076 | -0.0087 | -0.0119 | -0.0602 |  |
