# Supplementary material for: Causations of phylogeographic barrier of some rocky shore species along the Chinese coastline
Source: BMC Evol Biol. 2015 Jun 15;15:114. doi: 10.1186/s12862-015-0387-0 (PMC4465721; doi:10.1186/s12862-015-0387-0)
Supplement: Additional file 3: Table S3. — Pairwise genetic distance (ΦST) among locations and P-values of mitochondrial sequence COIII of Sargassum horneri are given in the lower and upper diagonals, respectively. Reference: Sargassum horneri, Hu et al. [36]. [file 12862_2015_387_MOESM3_ESM.docx]

## Additional file 3: Table S3. Pairwise genetic distance (ΦST) among locations and *P*-values of mitochondrial sequence COIII of *Sargassum horneri* are given in the lower and upper diagonals, respectively. Reference: *Sargassum horneri*, Hu *et al*. [36].

|  | **DL** | **DQ** | **QD** | **LX** | **NJ** | **FJ** | **NZ** |
| --- | --- | --- | --- | --- | --- | --- | --- |
| DL |  | 0.8079 | 0.1175 | 0.0007 | 0.0079 | 0.6404 | 0.0301 |
| DQ | -0.0258 |  | 0.3394 | 0.0008 | 0.0009 | 0.7461 | 0.0055 |
| QD | 0.0302 | 0.0043 |  | 0.0001 | 0.0000 | 0.4149 | 0.0001 |
| LX | 0.2374 | 0.2118 | 0.2083 |  | 0.0000 | 0.0705 | 0.0000 |
| NJ | 0.1147 | 0.1774 | 0.2768 | 0.6257 |  | 0.0040 | 0.0514 |
| FJ | -0.0287 | -0.0401 | 0.0003 | 0.1217 | 0.3118 |  | 0.0047 |
| NZ | 0.0867 | 0.1446 | 0.2264 | 0.5529 | 0.0849 | 0.2497 |  |
