# Supplementary figures and images for: Causations of phylogeographic barrier of some rocky shore species along the Chinese coastline
Source: BMC Evol Biol. 2015 Jun 15;15:114. doi: 10.1186/s12862-015-0387-0 (PMC4465721; doi:10.1186/s12862-015-0387-0)

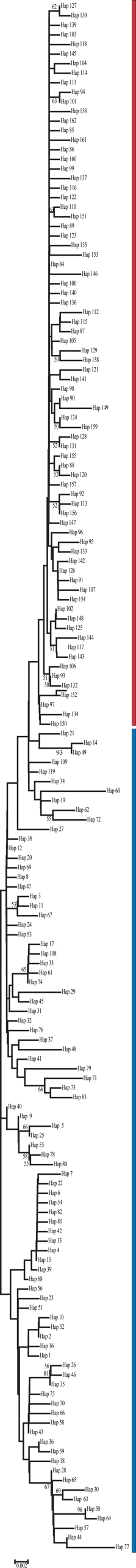

Supplement: Additional file 4: Figure S1. — Neighbour-joining tree of COI from Siphonaria japonica. Neighbour-joining tree constructed with mitochondrial DNA haplotype data from Siphonaria japonica. The classification of the two groups is given on the right. [file 12862_2015_387_MOESM4_ESM.pdf]

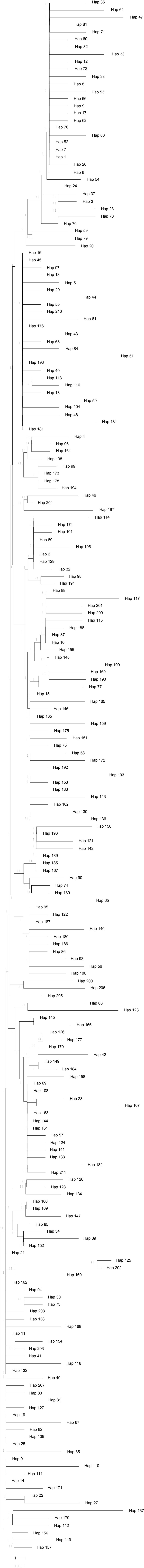

Supplement: Additional file 5: Figure S2. — Neighbour-joining tree of ITS from Siphonaria japonica. Neighbour-joining tree constructed with nuclear DNA haplotype data from Siphonaria japonica. [file 12862_2015_387_MOESM5_ESM.pdf]

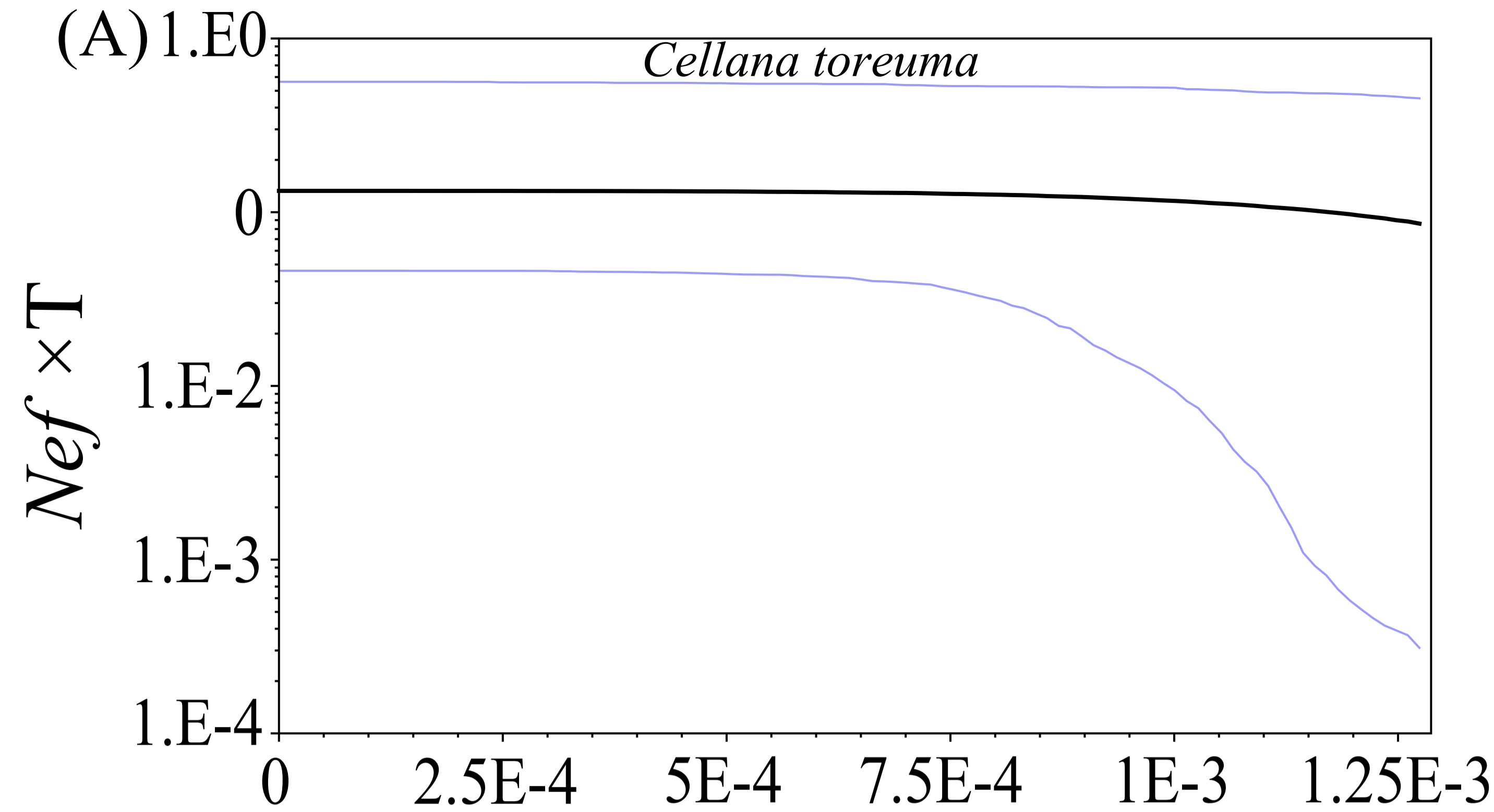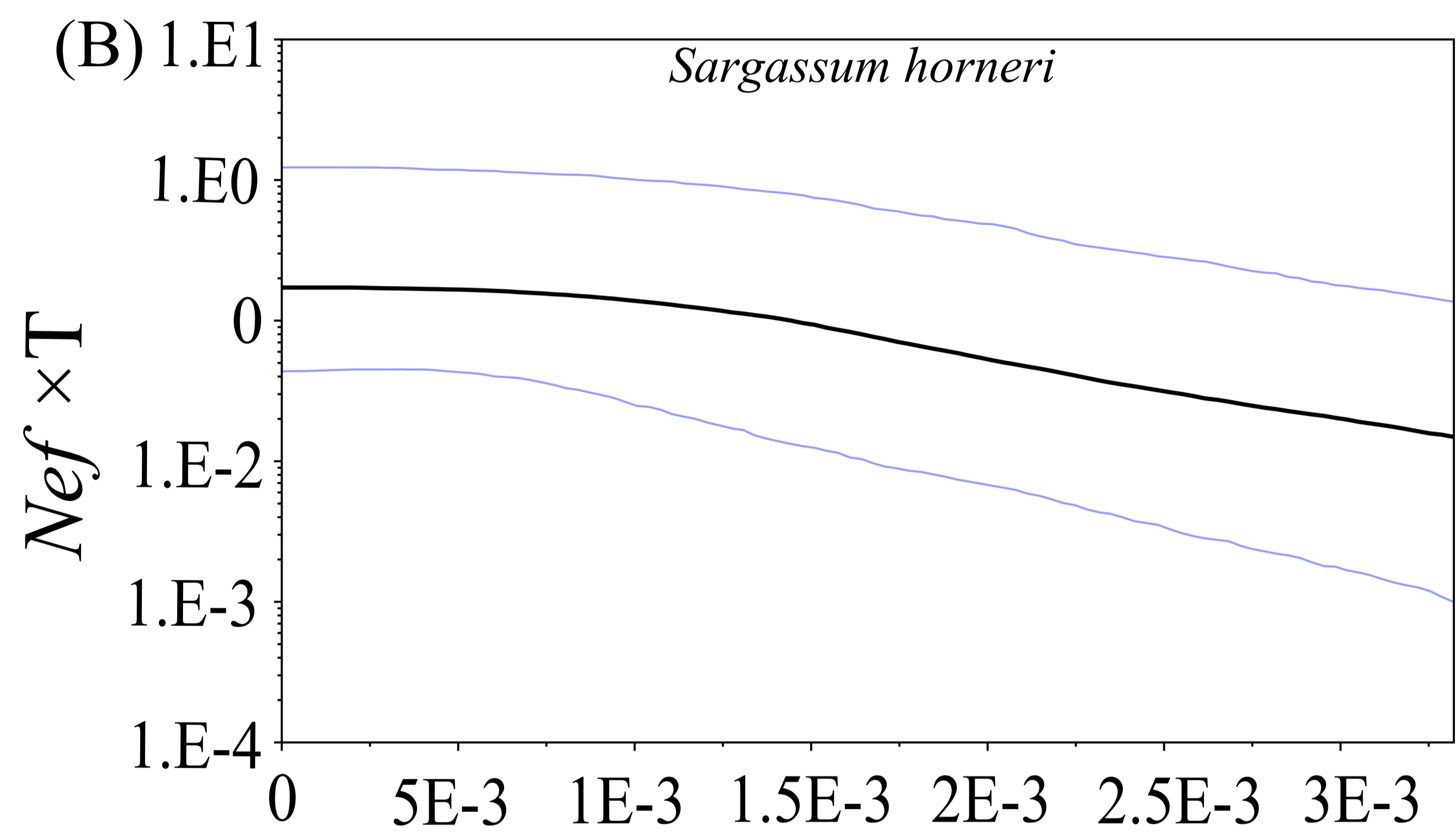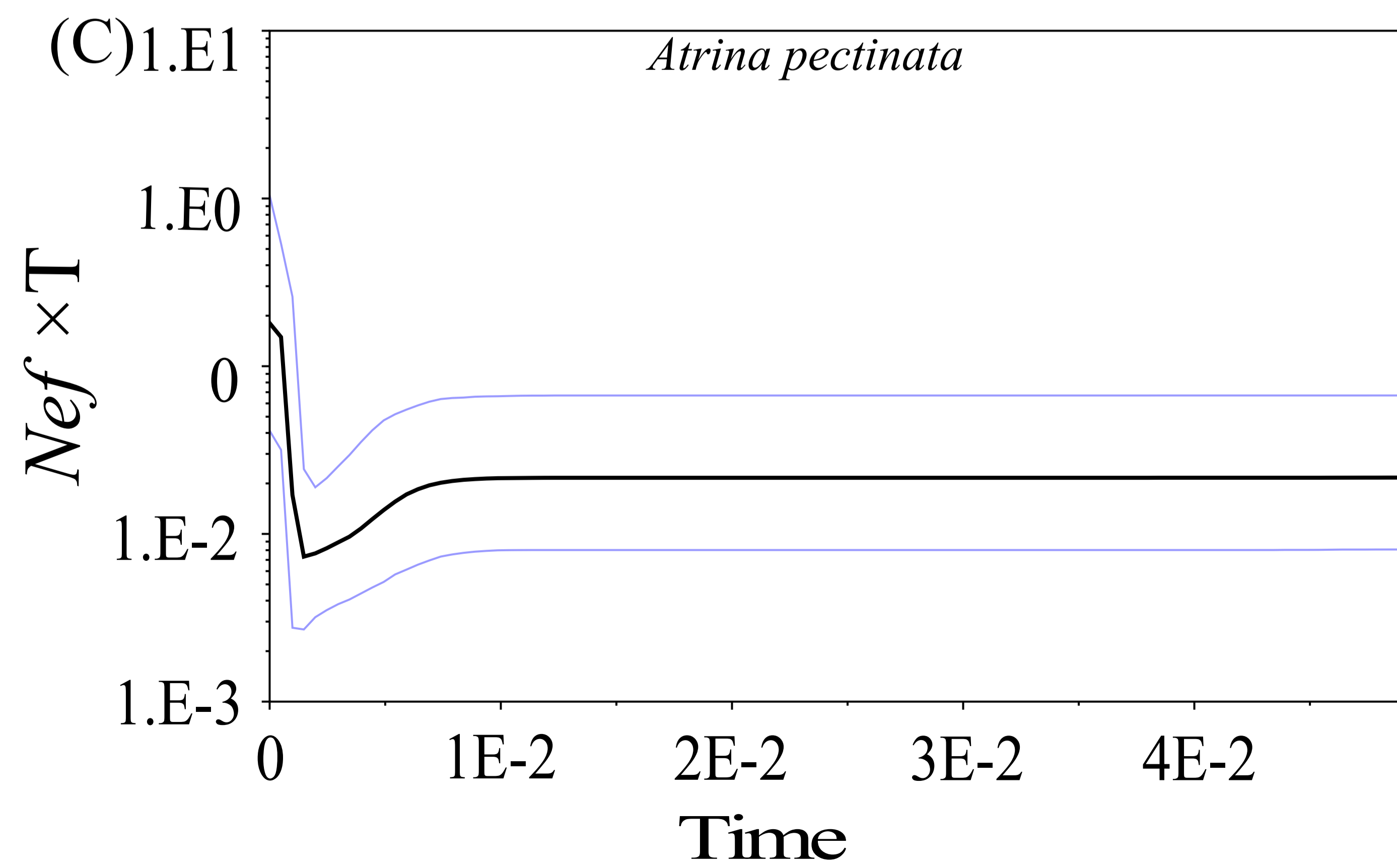

Supplement: Additional file 7: Figure S3. — Bayesian skyline plots (BSP) for Cellana toreuma (A), Sargassum horneri (B) and Atrina pectinata (C). The black line represents median population estimates; the upper and lower limits of light blue shading represent the 95 % confidence intervals. Reference: Cellana toreuma, Dong et al. [20]; Sargassum horneri, Hu et al. [36]; Atrina pectinata, Liu et al. [37]. [file 12862_2015_387_MOESM7_ESM.pdf]
