# Supplementary material for: Causations of phylogeographic barrier of some rocky shore species along the Chinese coastline
Source: BMC Evol Biol. 2015 Jun 15;15:114. doi: 10.1186/s12862-015-0387-0 (PMC4465721; doi:10.1186/s12862-015-0387-0)
Supplement: Additional file 6: Table S4. — A posterior simulation-based analogue of Akaike’s information reiteration through MCMC (AICM) test was used to compare demographic models for all four species. The estimated AICM scores of the posterior are listed in the third column for each species, and lower value indicates a better fit to the data. Boldface corresponds to the best fitting demographic model. The AICM comparisons were presented in the matrix of columns 5 to 7. The positive value represents the support for the one model over another. Reference: Cellana toreuma, Dong et al. [20]; Sargassum horneri, Hu et al. [36]; Atrina pectinata, Liu et al. [37]. [file 12862_2015_387_MOESM6_ESM.docx]

**Additional file 6: Table S4.** A posterior simulation-based analogue of Akaike’s information reiteration though MCMC (AICM) test was used to compare demographic models for all four species. The estimated AICM scores of the posterior are listed in the third column, and lower value indicates a better fit to the data. Boldface corresponds to the best fitting demographic model. The AICM comparisons were presented in the matrix of columns 5 to 7. The positive value represents the support for the one model the others. Reference: *Cellana toreuma*, Dong *et al*. [20]; *Sargassum horneri*, Hu *et al*. [36]; *Atrina pectinata*, Liu *et al.* [37].

| **Species** | **Model** | **AICM** | **S.E.** | **Comparison** | | |
| --- | --- | --- | --- | --- | --- | --- |
|  |  |  |  | **BSP** | **Constant size** | **Expansion** |
| *S. japonica* Northern group | BSP | 9182.9 | ±0.568 | - | -5156.2 | -5175.2 |
|  | Constant | 4026.7 | ±0.296 | 5156.2 | - | -19.0 |
|  | **Expansion** | 4007.7 | ±0.289 | 5175.2 | 19.0 | - |
| *S. japonica* Southern group | BSP | 15264.9 | ±12.296 | - | -11658.5 | -11559.9 |
|  | **Constant** | 3606.5 | ±0.343 | 11658.5 | - | 98.6 |
|  | Expansion | 3705.1 | ±0.454 | 11559.9 | -98.6 | - |
| *Cellana toreuma* | BSP | 2518.3 | ±0.617 | - | -572.7 | -558.5 |
|  | **Constant** | 1945.6 | ±0.048 | 572.7 | - | 14.2 |
|  | Expansion | 1959.8 | ±0.195 | 558.5 | -14.2 | - |
| *Sargassum horneri* | BSP | 6482.2 | ±0.365 | - | -4735.8 | -4731.9 |
|  | **Constant** | 1746.4 | ±0.098 | 4735.8 | - | 3.9 |
|  | Expansion | 1750.3 | ±0.178 | 4731.9 | -3.9 | - |
| *Atrina pectinata* | BSP | 11025.3 | ±3.52 | - | -5690.6 | -5701.1 |
|  | Constant | 5334.6 | ±0.337 | 5690.6 | - | -10.5 |
|  | **Expansion** | 5324.1 | ±0.321 | 5701.1 | 10.5 | - |
