# Supplementary material for: Causations of phylogeographic barrier of some rocky shore species along the Chinese coastline
Source: BMC Evol Biol. 2015 Jun 15;15:114. doi: 10.1186/s12862-015-0387-0 (PMC4465721; doi:10.1186/s12862-015-0387-0)
Supplement: Additional file 8: Table S5. — The effective population size (Θ) and the effective immigration rates (M) with 97.5 % credibility intervals in parentheses were estimated using the program MIGRATE for each species. All sampling sites of each species are divided into Yellow Sea (YS) group and East plus South China Seas (ESCS) group according to the Yangtze River Estuary. Reference: Cellana toreuma, Dong et al. [20]; Sargassum horneri, Hu et al. [36]; Atrina pectinata, Liu et al. [37]. [file 12862_2015_387_MOESM8_ESM.docx]

**Additional file 8: Table S5.** The effective population size (*Θ*) and the effective immigration rates (*M*) with 97.5% credibility intervals in parentheses were estimated using the program MIGRATE for each species. All sampling sites of each species are divided into Yellow Sea (YS) group and East plus South China Seas (ESCS) group according to the Yangtze River Estuary. Reference: *Cellana toreuma*, Dong *et al*. [20]; *Sargassum horneri*, Hu *et al*. [36]; *Atrina pectinata*, Liu *et al.* [37].

| **Species (locus)** | **Grouping** | **Effective population size (*Θ*)** | **Immigration rate (*M*_ESCS-YS_)** | **Immigration rate (*M*_YS-ESCS_)** |
| --- | --- | --- | --- | --- |
| *Siphonaria japonica* (COI) | YS | 0.07319 (0.05580-0.09607) | 16.3 (0.0-84.7) |  |
|  | ESCS | 0.09810 (0.08207-0.10000) |  | 143.7 (70.0-249.3) |
| *Siphonaria japonica* (ITS) | YS | 0.08633 (0.05600-0.15253) | 534.3 (328.0-906.7) |  |
|  | ESCS | 0.09540 (0.07533-0.11973) |  | 0.3 (0.0-38.0) |
| *Cellana toreuma* (COI) | YS | 0.00143 (0.00000-0.00333) | 8686.7 (933.3-18040.0) |  |
|  | ESCS | 0.01630 (0.00800-0.02707) |  | 8953.3 (5426.7-13266.7) |
| *Sargassum horneri* (COIII) | YS | 0.47767 (0.05800-0.88600) | 2218.3 (1033.3-4096.7) |  |
|  | ESCS | 0.01033 (0.00000-0.02133) |  | 255.0 (0.0-1480.0) |
| *Atrina pectinata* (COI) | YS | 0.03383 (0.01768-0.04967) | 190.0 (0.0-713.3) |  |
|  | ECS | 0.33617 (0.12233-0.50010) |  | 4036.7 (1920.0-7240.0) |
